# Aripirazole augmentation in clozapine-associated obsessive-compulsive symptoms in schizophrenia

**DOI:** 10.1186/1744-859X-12-40

**Published:** 2013-12-12

**Authors:** Gül Eryılmaz, Gökben Hızlı Sayar, Eylem Ozten, Işıl Gögcegöz Gül, Oğuz Karamustafalıoğlu

**Affiliations:** 1 Department of Psychiatry, Uskudar University, Neuropsychiatry Istanbul Hospital, Alemdag Caddesi Site Yolu No:29, Umraniye, Istanbul, Turkey

**Keywords:** Adjunctive therapy, Aripiprazole, Clozapine, Obsessive compulsive symptoms

## Abstract

**Objective:**

Patients with schizophrenia often experience comorbid obsessive-compulsive symptoms. Within these patients, a significant subgroup developed secondary obsessive-compulsive symptoms during treatment with clozapine.

**Method:**

In this paper, we report on four cases in which adjunctive therapy with aripiprazole was tested to alleviate obsessive-compulsive symptoms in schizophrenia.

**Results:**

All four patients had a significant improvement in obsessive-compulsive symptoms. The combination of clozapine and aripiprazole was well tolerated.

**Conclusion:**

This case series demonstrates the clinical efficacy of aripiprazole adjunctive therapy with clozapine in schizophrenic patients with comorbid obsessive-compulsive symptoms. Larger-sampled and controlled studies are required in order to test and confirm these observations.

## Introduction

The incidence of obsessive-compulsive symptoms (OCS) in schizophrenia had been reported as 20–30% [1]. Publications related to exacerbation of the OCS in patients with schizophrenia by atypical antipsychotics focused on the mechanism of action of these drugs [2].

The striatal 5-HT2 antagonism of new-generation antipsychotics may result in the emergence of OCS via an overactive dopaminergic system. The weak D2 receptor-blocking properties of clozapine may be also accounted for the onset of OCD-like symptoms [3]. Temporary OCS is associated with the dose of clozapine reported in the literature [4]. A total of 55 patients are included in 30 reports between 1990 and 2002, focusing on atypical antipsychotic treatment-induced OCS or exacerbation of preexisting OCS in patients with schizophrenia. Of these patients, 30 developed OCS with the use of clozapine, 16 with risperidone, 8 with olanzapine, and 1 with quetiapine [5]. In a study of 50 patients with a diagnosis of schizophrenia treated with clozapine, 10 of the subjects developed OCS after clozapine treatment and 9 patients had an increase in preexisting OCS [6]. In treatment resistant of schizophrenia, the first-line treatment option is clozapine but with the dose escalation, the risk of triggering OCS and need for adjunctive therapy arises [7,8].

This study summarizes the courses of four patients with schizophrenia who experienced OCS under treatment with clozapine so dose escalation of clozapine could not be done and need for augmentation therapy arises. The effects of aripiprazole and clozapine combination on OCS and psychotic symptoms are presented.

## Cases

### Case 1

A 51-year-old, Caucasian, male patient, diagnosed with schizophrenia was admitted following an exacerbation of OCS. He had contamination obsessions and cleaning compulsions for 10 years. He had persecution delusions. He was thinking that he was followed by police. He was hoarding garbage. He was brought to the emergency room by his family. Both the patient and his family reported that he had benefitted most from clozapine treatment. He was on clozapine 100 mg/day for 4 years. However, when the dose of clozapine was increased to a dose of 150 mg/day, an increase in OCS was observed. His family reported that a year ago after he had 10 sessions of electroconvulsive therapy (ECT), his psychotic and OCS decreased; but for the last 5–6 months, there was an exacerbation in psychotic and obsessive symptoms. Patient was hospitalized. During admission, his Positive and Negative Syndrome Scale (PANSS), Brief Psychiatric Rating Scale (BPRS), and Yale–Brown Obsessive Compulsive Scale (Y-BOCS) scores were 151, 74, and 40, respectively.

A 30-mg/day dose of aripiprazole was added on to his ongoing 100-mg/day dose of clozapine treatment. After 2 weeks of combination treatment with 100 mg/day clozapine and 10 mg/day aripiprazole, plasma levels of both drugs were measured to be low. Clozapine dose increased to 250 mg/day and aripiprazole doze increased to 30 mg/day gradually. At the end of the sixth week, during discharge, his PANSS, BPRS, and Y-BOCS scores were 50, 20, and 20, respectively.

### Case 2

A 28-year-old, Caucasian, male patient, with a diagnosis of residual schizophrenia, treated for 2 years with clozapine 100 mg/day was admitted because of the worsening of his psychotic symptoms. His OCS first was seen at age of 13. He had control and symmetry obsessions and compulsions. After 5 years of OCS, he had psychotic symptoms. He reported that he could not leave the house for the last 2 years, due to his persecution delusions, skepticism, and resentment. He was on a treatment of clozapine 100 mg/day for 2 years.

He was hospitalized with a dose of 10 mg/day aripiprazole added on to his ongoing 100-mg/day clozapine treatment. The plasma level of clozapine and norclozapine were lower than therapeutic level at the admission. At the end of the second week, clozapine dose increased to 200 mg/day and aripiprazole to 30 mg/day gradually. The clozapine, norclozapine, aripiprazole doses and the clinical rating scale scores are given at Table 1. During discharge, his scores of PANSS was 49 (78 at admission), BPRS was 27 (39 at admission), and Y-BOCS was 15 (40 at admission).

**Table 1:** The clinical rating scale scores, doses, and plasma concentrations of clozapine and aripiprazole

	**1st day**	**2nd week**	**3rd week**	**4th week**	**PANSS**	**Y-BOCS**
Case 1: age 51, male	100 mg/day clozapine	100 mg/day clozapine	250 mg/day clozapine	250 mg/day clozapine	Baseline	Baseline
	(90.7 ng/mL)	(96.2 ng/mL)	(215 ng/mL)	(132.5 ng/mL)	151	40
	+	+	+	+	4th week	4th week
	30 mg/day aripiprazole	30 mg/day aripiprazole	30 mg/day aripiprazole	30 mg/day aripiprazole	50	20
		(222.2 ng/mL)	(300.7 ng/mL)	(322.8 ng/ml)		
Case 2: age 28, male	100 mg/day clozapine	200 mg/day clozapine	200 mg/day clozapine	100 mg/day clozapine	Baseline	Baseline
	(142.8 ng/mL)	(198.0 ng/mL)	(332.7 ng/mL)	(232 ng/mL)	78	40
	+	+	+	+	4th week	4th week
	30 mg/day aripiprazole	30 mg/day aripiprazole	30 mg/day aripiprazole	30 mg/day aripiprazole	49	15
		(100.78 ng/mL)	(363.51 ng/mL)	(368.23 ng/ml)		
Case 3: age 34, male	100 mg/day clozapine	150 mg/day clozapine	150 mg/day clozapine	100 mg/day clozapine	Baseline	Baseline
	(52.7 ng/mL)	(291.4 ng/mL)	(749.1 ng/mL)	(546.2 ng/mL)	141	40
	+	+	+	+	4th week	4th week
	30 mg/day aripiprazole	30 mg/day aripiprazole	15 mg/day aripiprazole	15 mg/day aripiprazole	50	20
		(787 ng/mL)	(243 ng/mL)	(310 ng/mL)		
Case 4: age 27, male	150 mg/day clozapine	200 mg/day clozapine	150 mg/d clozapine	100 mg/d clozapine	Baseline	Baseline
	(142 ng/mL)	(332 ng/mL)	(241.7 ng/mL)	(203.4 ng/mL)	159	30
	+	+	+	+	4th week	4th week
	30 mg/day aripiprazole	30 mg/day aripiprazole	30 mg/day aripiprazole	30 mg/day aripiprazole	46	10
		(353 ng/mL)	(358.4 ng/mL)	(347.8 ng/mL)		

### Case 3

A 34-year-old, Caucasian, male patient with a diagnosis of paranoid schizophrenia for 14 years was admitted for psychiatric examination with complaints of repeating some words and washing hands frequently. He was on clozapine 100 mg/day treatment for 2 years. He was hospitalized due to exacerbation of psychotic and OCS.

A 10-mg/day dose of aripiprazole was added on to his ongoing 100-mg/day clozapine treatment. At the end of the first week, clozapine dose increased to 150 mg/day and aripiprazole dose to 30 mg/day gradually. The plasma clozapine and norclozapine levels were over therapeutic level as 746.1 and 519.1 ng/mL at the end of the third week, so clozapine dose decreased to 100 mg/day. The clozapine andaripiprazole doses, plasma drug levels, and the clinical rating scale scores are given at Table 1. During discharge, his scores of PANSS was 50 (141 at admission), BPRS was 20 (74 at admission), and YBOCS was 20 (40 at admission).

### Case 4

A 27-year-old, Caucasian, male patient, diagnosed with substance-induced psychotic disorder, was hospitalized due to exacerbation of psychotic symptoms despite clozapine dose of 150 mg/day for two years. His family reported exacerbation of obsessive-compulsive symptoms, such as contamination obsessions and frequent handwashing, for 3 months. Clozapine and norclozapine plasma levels were lower than therapuetic level on admission. a 15-mg/day aripiprazole was added on and clozapine dose increased to 200 mg/day. At the end of the first week, aripiprazole dose was increased to 30 mg/day. Plasma clozapine and norclozapine levels were found to be higher than therapeutic level with a clozapine dose of 200 mg/day, and clozapine dose decreased to 100 mg/day. The clozapine and aripiprazole doses, plasma drug levels, and the clinical rating scale scores are given in Table 1. At the end of the fourth week, during discharge, his scores of PANSS was 46 (159 at admission), and YBOCS was 10 (30 at admission).

## Discussion

Schizophrenia is a chronic, severe, and disabling brain disorder and affects roughly 1% of the population. Twenty to thirty percent of the schizophrenic patients had a partial response or no response, although they have adequate dose and duration for the treatment of typical and atypical antipsychotic drugs [9]. Here, we report four patients with a diagnosis of schizophrenia who benefited the clozapine treatment but due to the emergence of OCS dose escalation of clozapine could not be done. They were all treatment-resistant schizophrenic patients and clozapine was the only effective medication these patients responded to so far.

Although clozapine is a first-line antipsychotic treatment in patients with treatment-resistant schizophrenia, 18–44% of patients who fail to respond to monotherapy of clozapine need augmentation with a second antipsychotic [10]. Various researchers reported the remission of schizophrenia psychosis and strong reduction of obsessive-compulsive disorder after adding clozapine to aripiprazole [11,12]. The choice of aripiprazole as an adjunctive treatment was suggested because of its mechanism of action [13]. The partial dopaminergic and serotonergic agonist aripiprazole *per se* was associated with an inherent antiobsessive potency. Bachmann et al. included 15 patients with schizophrenia from a child and adolescent psychiatric department. Patients had been under clozapine treatment, followed by aripiprazole augmentation. They concluded that in adolescents with schizophrenia, aripiprazole augmentation of clozapine treatment might be an effective therapeutic strategy [14]. Akdag et al. reported that aripiprazole augmentation in 11 patients with a diagnosis of schizophrenia treated for at least 6 months with clozapine resulted in a marked improvement in 7 patients, with a diminish in BPRS scores. Aripiprazole augmentation did not result in any additional side effects [15]. Chanachev et al. reported on two cases in which augmentation with aripiprazole had a beneficial impact on anxiety [16]. In a study examining aripiprazole treatment in patients with comorbid schizophrenia and OCS that did not meet the full criteria for obsessive-compulsive disorder, results suggest that aripiprazole monotherapy can modestly improve the outcome for some schizophrenia patients with obsessive-compulsive symptoms [17]. In a case study, a patient with paranoid schizophrenia developed OCS during long-term treatment with olanzapine at 20 mg/day over a period of 10 years. The combination with aripiprazole (15 mg/day) over a period of 12 weeks resulted in a marked improvement of OCS and some further improvement of the psychotic symptoms. This observation points toward an antiobsessive potency of aripiprazole in combination with olanzapine, quite similar to approaches involving clozapine [18].

It has been proposed that antiserotonergic second-generation antipsychotics might induce OCS. Schirmbeck et al. reported that OCS severity correlated with a dosage of clozapine and duration of treatment [19]. It was reported that reducing the dose of ‘pro-obsessive’ clozapine by using combinations with mainly dopaminergic antipsychotics might be helpful [20]. Englisch et al. reported seven patients with schizophrenia developed secondary OCS during treatment with clozapine. A marked reduction of obsessions and significant improvements of compulsions observed after aripiprazole add-on treatment, pointing toward an antiobsessive potency of aripiprazole [21].

The combination of clozapine and aripiprazole had several benefits: reduction of clozapine dose, improvement of positive and negative symptoms, and reduction of OCS. This augmentation strategy was well tolerated with ne epileptical seizures, agranulocytosis, extrapiramidal side effects, or cognitive side effects.

This case report's limitations include the small sample size, non-controlled and inhomogeneous sample, possible affective course, different nature of schizophrenic symptoms, and the high doses of aripiprazole. The rapid improvement associated to the adjunctive treatment with aripiprazole may also be favored by a mood enhancement. Aripiprazole is also a 5-H1A partial agonist, which may concur to pro-cognitive, anti-OCD, and mood-ameliorating effects. Also, the history of previous response to ECT may also indirectly suggest an affective course. Unexplained relapses of the patients during clozapine treatment may indicate poor medication adherence.

## Conclusion

Patients suffering from clozapine-induced OCS deserve close attention. Owing to superior antipsychotic potency of clozapine, clinicians may not want to switch from clozapine to another antipsychotic. It might be helpful to reduce the dose of ‘pro-obsessive’ clozapine by using a combination with aripiprazole. Here, our findings suggest that the adjunctive use of aripiprazole with clozapine represents a new therapeutic strategy in cases of schizophrenic patients who continue to present clinically significant obsessive-compulsive symptoms. This finding can be proved by controlled studies; it would be of major clinical interest.

This case report suggests that a combination of clozapine and aripiprazole may optimize treatment efficacy and tolerability in schizophrenic patients with comorbid OCS. In the future, the proposed strategy should be further evaluated in prospective controlled trials.

### Consent

Written informed consent was obtained from the patients for publication of this case report and any accompanying images.

## Abbreviations

OCS: Obsessive compulsive symptoms.

## Competing interests

The authors declare that they have no competing interests.

## Authors' contributions

GE and EO analyzed and interpreted the patient data. GHS wrote the manuscript. IGG searched literature. OK was a major contributor in the writing of the manuscript. All authors read and approved the final manuscript.
